# Modeling zero-lag synchronization of dorsal horn neurons during the traveling of electrical waves in the cat spinal cord

**DOI:** 10.1002/phy2.21

**Published:** 2013-07-08

**Authors:** Hideyuki Kato, Carlos A Cuellar, Rodolfo Delgado-Lezama, Pablo Rudomin, Ismael Jimenez-Estrada, Elias Manjarrez, Claudio R Mirasso

**Affiliations:** 1 Instituto de Física Interdisciplinar y Sistemas Complejos (IFISC, UIB-CSIC), Campus Universitat de les Illes BalearsE-07122, Palma de Mallorca, Spain; 2 Departamento de Fisiología, Biofísica y Neurociencias CINVESTAVAv. IPN 2508, Col. San Pedro Zacatenco, CP 07360, México; 3 Instituto de Fisiología, Benemérita Universidad Autónoma de Puebla14 Sur 6301, Apartado Postal 406, Col. San Manuel, Puebla, Pue, CP 72570, México

**Keywords:** Dorsal horn neurons, scratching, traveling waves, central pattern generator

## Abstract

The first electrophysiological evidence of the phenomenon of traveling electrical waves produced by populations of interneurons within the spinal cord was reported by our interdisciplinary research group. Two interesting observations derive from this study: first, the negative spontaneous cord dorsum potentials (CDPs) that are superimposed on the propagating sinusoidal electrical waves are not correlated with any scratching phase; second, these CDPs do not propagate along the lumbosacral spinal segments, but they appear almost simultaneously at different spinal segments. The aim of this study was to provide experimental data and a mathematical model to explain the simultaneous occurrence of traveling waves and the zero-lag synchronization of some CDPs.

## Introduction

The lumbar spinal cord exhibits spontaneous electrical activity produced by neuronal ensembles located in the dorsal horn. Such spontaneous activity was defined with the term “spontaneous cord dorsum potentials (CDPs).” These CDPs were first recorded by Bremer (1941) and Ten Cate (1950). Mark and Gasteiger (1953) suggested that the CDPs were generated by intrinsic spinal mechanisms. Later, (Gasteiger and Ichikawa 1963; see also Gasteiger 1969) found that the spontaneous potentials appeared to be largest in the dorsal gray matter, and that they were amplified after spinalization. In subsequent studies by Manjarrez et al. (2000, 2003), a detailed analysis on the origin and physiological role of these electrical potentials was performed. These authors reported that large amplitude CDPs were generated by the synchronous activation of a population of dorsal horn neurons (Rexed's laminae III–VI) that respond monosynaptically to stimulation of low-threshold cutaneous and Ib muscle afferents. Moreover, in an anesthetized cat, the CDPs lasting 40–60 msec have characteristic low-frequency components (3–20 Hz). Based on these results, it was assumed that CDPs were generated by the spontaneous activity of a population of dorsal horn neurons distributed along several lumbosacral spinal segments which fired action potentials in a highly synchronized manner (Manjarrez et al. 2002; Manjarrez et al. 2003). A subsequent study from Garcia et al. 2004, showed that the acute section of both the intact sural and superficial peroneal nerve increased the variability of the spontaneous nCDPs without affecting their intersegmental coupling. However, a lesion comprising the left dorsal quadrant between segments L5 and L6 produced a reduction in the synchronization between spontaneous CDPs recorded from the L5 and L6 segments in both sides of the spinal cord (Rodriguez et al. 2011). These findings are consistent with the fact that strengthening and weakening of coupling the neuronal networks is a particular feature of many circuits in the spinal cord (for review, see Jankowska et al. 2007). They are also consistent with previous evidence of commissural interneurons in the spinal cord, which could share neuronal circuits with neurons producing spontaneous CDPs (Jankowska et al. 2009).

Recently, Chávez et al. (2012) showed the occurrence of two different spontaneous CDPs, one purely negative and the other a negative–positive potential. The authors indicated that the neuronal assemblies producing both potentials had a similar longitudinal distribution throughout several spinal segments (L4–S1). A new classification algorithm of CDPs was reported (Vidaurre et al. 2012), thus allowing a faster classification of CDPs purely negative or purely negative–positive. These authors suggested that all the classes of spontaneous CDPs are produced by the activation of tightly coupled arrays of neurons that fire in a highly synchronized manner. However, little is known about the circuitry and electrophysiological properties of these dorsal horn neurons; furthermore, there are no models capable of explaining its spontaneous activity.

In recent years, Cuellar et al. (2009) and Perez et al. (2009) described with experiments and modeling the occurrence of a traveling electrical wave (termed sinusoidal CDPs) that travels rostrocaudally from L4 to S1 spinal segments during fictive scratching. According to these authors the sinusoidal CDPs represent the activity of the central pattern generator (CPG) and its followers. An interesting observation and further analysis derived from the study of Cuellar et al. (2009) is that the spontaneous CDPs superimposed on the sinusoidal electrical waves did not longitudinally propagate, but appeared simultaneously at several spinal segments, with practically no delay, and were not correlated with any scratching phase (Fig. 1A from Cuellar et al. 2009, illustrates sharp negative peaks in the recordings 3–5, before, during, and after the scratching episode).

The aim of this study was to provide experimental and theoretical basis about this observation in terms of the zero-lag synchronization framework (Vicente et al. 2008). Our study presents evidence that the spontaneous CDPs and the sinusoidal electrical traveling waves recorded on the cat spinal cord can be modeled within a framework of spinal cord neuronal models. We conjecture that the spontaneous potentials are mainly related to sensory processes, which exhibit zero-lag synchronization; however, the sinusoidal CDPs are associated with motor responses which exhibit longitudinal wave propagation.

## Methods

### Surgical procedures

The data presented in this study were obtained from ten experiments that were carried out throughout several experimental protocols performed in the laboratory, the latter with the intention of taking advantage of every experimental subject and reduce the number of slaughtered animals. The methods for surgical procedures were described elsewhere (Cuellar et al. 2009). Guidelines contained in the Mexican Norm for Care and Use of Animal for Scientific Purposes, NOM-062-ZOO-1999 (SAGARPA, Del. Benito Juárez, México), were strictly followed. Briefly, adult cats (2.0–3.5 kg) were anesthetized with isoflurane (2%) in a mixture with 100% oxygen. Atropine (0.05 mg/kg) and dexamethasone (2 mg/kg) were administered at the beginning of surgery. Arterial blood pressure was monitored by means of a cannula inserted in the carotid artery. This cannula allows the administration of a bicarbonate (100 mmol/L) and glucose (5%) solution throughout the experiment at a rate of 5 mL/h. The radial vein was also cannulated for the administration of dextran and saline solutions when it was necessary to maintain the animal's blood pressure between 80 and 120 mmHg. The lumbosacral (L4–S1) and cervical (C1–C2) spinal segments were exposed by performing a laminectomy, and then the dura mater was removed. The following muscle nerves were dissected free in both hindlimbs: tibialis anterior (TA), lateral gastrocnemius plus soleus (LGS), and medial gastrocnemius (MG). After surgical procedures, the animal was transferred to a metal stereotaxic frame, and pools were constructed around the exposed tissues, which were subsequently filled with mineral oil to avoid desiccation, and the dissected nerves were prepared for recording. Then the animals were decerebrated with a mechanical transection at the precollicular–postmammillary level and removal of all rostral brain tissue. After this procedure, the anesthesia was discontinued and the animals were paralyzed with pancuronium bromide (Pavulon; Organon Mexicana, México City, D.F., Mexico) and artificially ventilated. The temperature of the animals was kept constant (37°C) by a heating pad and radiant heat lamp.

### Electrophysiological recordings

Fictive scratching was induced by tactile stimulation of the pinna or other scratch receptive fields, after the application of D-tubocurarine (0.1%, 14 mmol/L) on the dorsal surface of the C1–C2 segments. Electrical field potentials recorded on the surface of the spinal cord (spontaneous activity and fictive scratching) were monopolarly recorded in direct (DC) or alternating current (AC) mode on a Synamps EEG amplifier (NeuroScan, Compumedics, Charlotte, NC). The cut-off frequency of band-pass filters was set at DC–500 Hz, 0.05–500, or 1–500 Hz. We employed a multielectrode system composed by 30 Ag–AgCl electrodes (200 μm diameter) positioned on the dorsal surface of the lumbosacral (L4–S1) spinal cord against an indifferent electrode placed on the paravertebral muscles for the recording of the spontaneous CDPs in left and right spinal segment locations (further details in Manjarrez et al. 2005). AC amplifiers (Grass, Astro-Med, West Warwick, RI) and the Digidata system (Molecular Devices, Sunnyvale, CA) were used to record the electrical activity (band pass, 0.05 Hz–30 kHz) from the dissected flexor and extensor motor nerves. The sampling rate in all recordings was 10 kHz. At the end of each experiment the animal was killed with an overdose of pentobarbital and perfused with 10% Formalin.

### Raster plot displays

**Figure 1:**
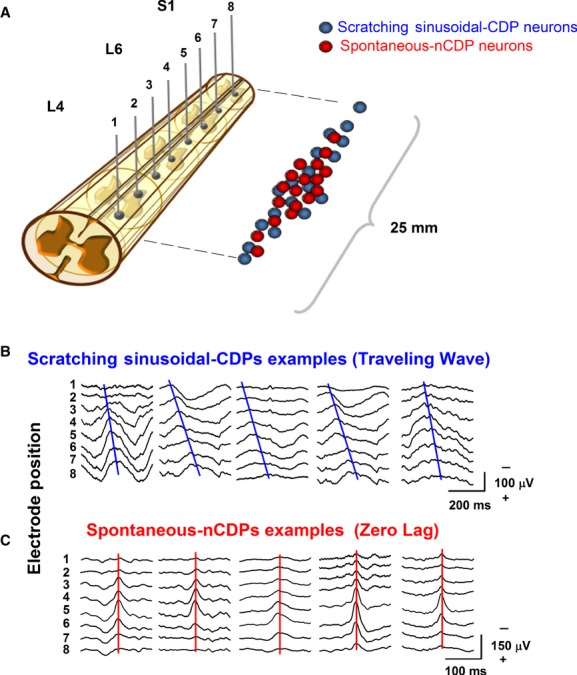
Electrical field potentials recorded in the surface of the spinal cord. (A) Spontaneous nCDPs and sinusoidal CDPs were analyzed taken one single column (eight electrodes) of the multielectrode array. The color balls represent two different populations of neurons; the red ones represent the neurons producing the spontaneous activity and the blue ones the neurons belonging to the CPG, thus generating the sinusoidal CDPs during fictive scratching. (B) Representative traces of the sinusoidal CDPs occurring during the fictive scratching. Note the phase gradient denoted by the blue line. (C) Representative traces of the spontaneous CDPs. Note the zero lag denoted by the red line.

In order to analyze the intersegmental occurrence of spontaneous and sinusoidal CDPs, we measured the occurrence time of the negative peak in a multielectrode column; then the values were time locked to zero (see for example Fig. 1B and C). Raster plot displays were constructed with the time values and the electrode position. For clarity in all the data presented, the electrode numbers were reassigned from 1 (more rostral electrode) to 8 (more caudal electrode).

## Results

### Experimental data

The intersegmental occurrence of both types of potentials, spontaneous CDPs and sinusoidal CDPs, is illustrated in Figure 1. The scheme in the Figure 1A shows for clarity just one electrode column (eight elements) of the multielectrode array described in the Methods section. The hypothesis is also sketched representing the intermingled distribution of the neurons generating the spontaneous CDPs (red balls) and the neurons belonging to the CPGs (blue balls). Figure 1A and B shows representative recordings of the scratching sinusoidal CDPs and the spontaneous CDPs. Each column of eight traces corresponds to an individual experiment (from a total of five experiments). For the case of the sinusoidal CDPs (Fig. 1B), the blue line indicates the peak of the negative component in the sinusoidal CDPs, represented by a traveling wave that sweeps rostrocaudally the lumbar spinal cord from the L4 to the S1 segments. On the other hand, spontaneous CDPs are synchronized (zero lag) as is indicated by the red line pointing out the peak of the negative component in spontaneous CDPs, independently if they were pure negative or negative–positive potentials (Fig. 1C). It is observed that the peak amplitude distribution of both potentials, sinusoidal and spontaneous CDPs, is similar to a bell shape, that is, the maximal amplitude occurs at the central electrodes and the minimal amplitude appears in the more rostral and caudal segments. Note the differences in timescale.

**Figure 2:**
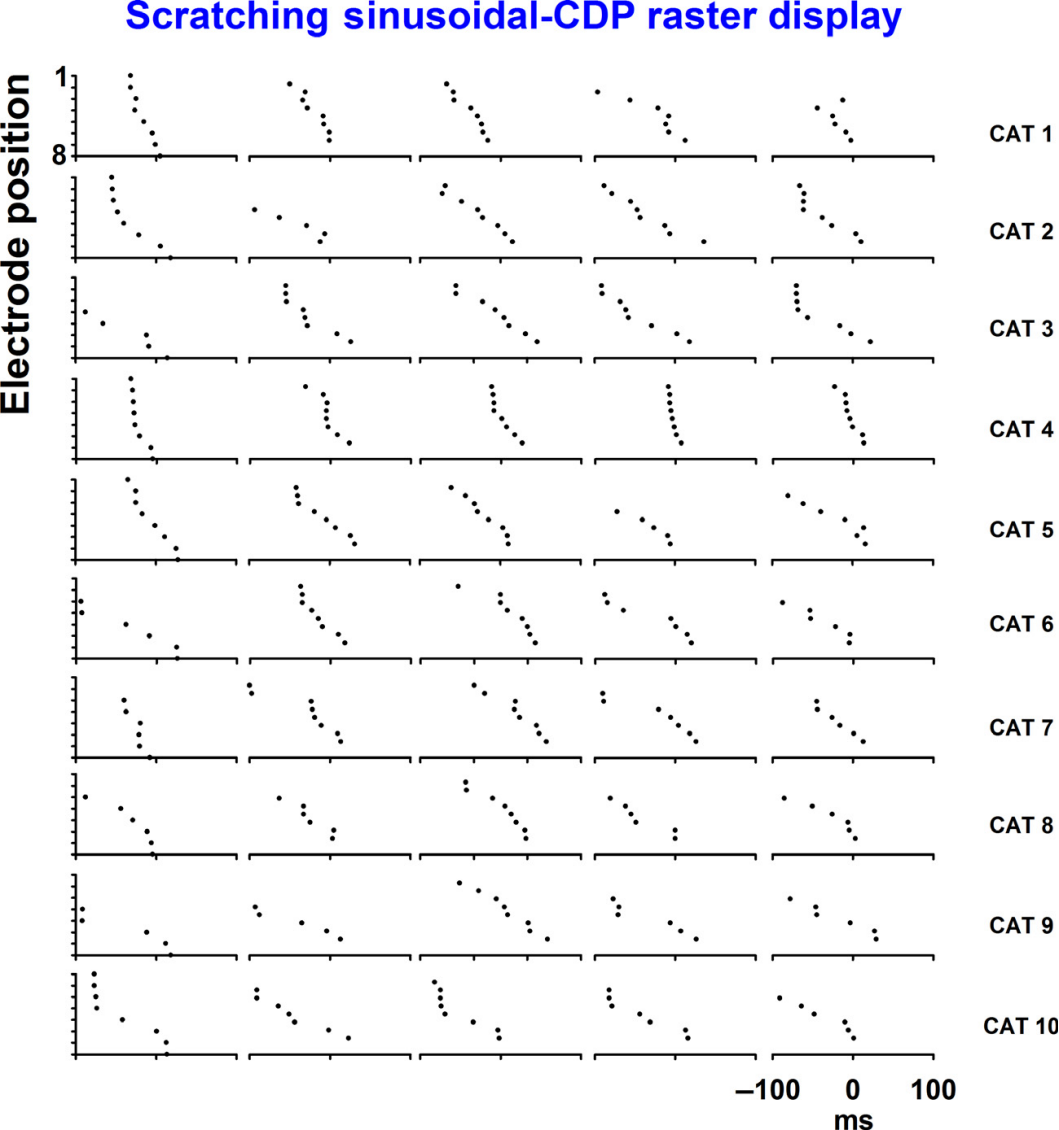
Scratching sinusoidal CDPs raster display. Five scratching cycles are shown for ten individual experiments. Note the rostrocaudal propagation and the variability in the phase gradient.

**Figure 3:**
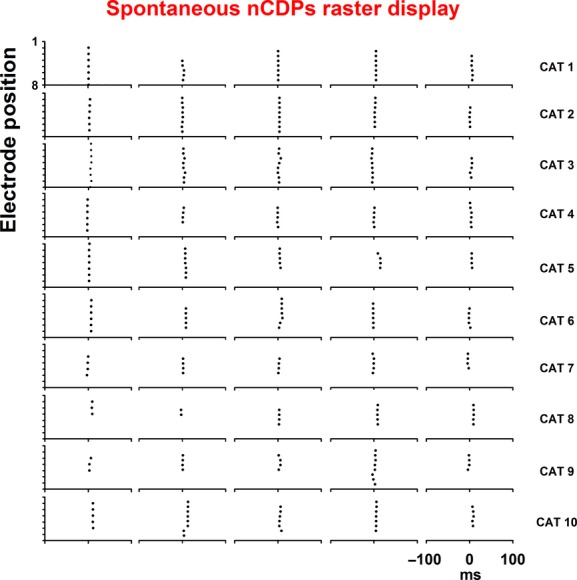
Spontaneous nCDPs raster display. Five spontaneous nCDPs are shown for ten individual experiments. Note the zero-lag synchronization across the potentials with minimal nonsignificant deviations.

In a previous study a gradient phase analysis related to the sinusoidal CDPs during fictive scratching was used to describe the propagation of the sinusoidal CDPs (Cuellar et al. 2009). Another qualitatively way to illustrate the propagation of this traveling wave is by means of a peak raster display as shown in Figure 2. The rows correspond to ten individual experiments and each column shows the occurrence of a scratching cycle (five cycles analyzed per experiment). Clearly, the gradient phase exhibits a rostrocaudal propagation with some differences in the range of time. It can also be observed, for example, that in some scratching cycles, even in the same animal, the gradient phase is not constant (traveling wave velocity), but the rostrocaudal propagation always remains. The same analysis based on raster display is presented in Figure 3 for the spontaneous CDPs recorded in the same series of experiments as before. Each point in the raster plot represents one spontaneous CDP recorded at several segments of the lumbosacral spinal cord. Note the remarkable synchronization between the simultaneously recorded spontaneous CDP. Although there are some deviations from the zero lag, the temporal variation is not significant, compared with the variation exhibited in Figure 2.

### Modeling

**Figure 4:**
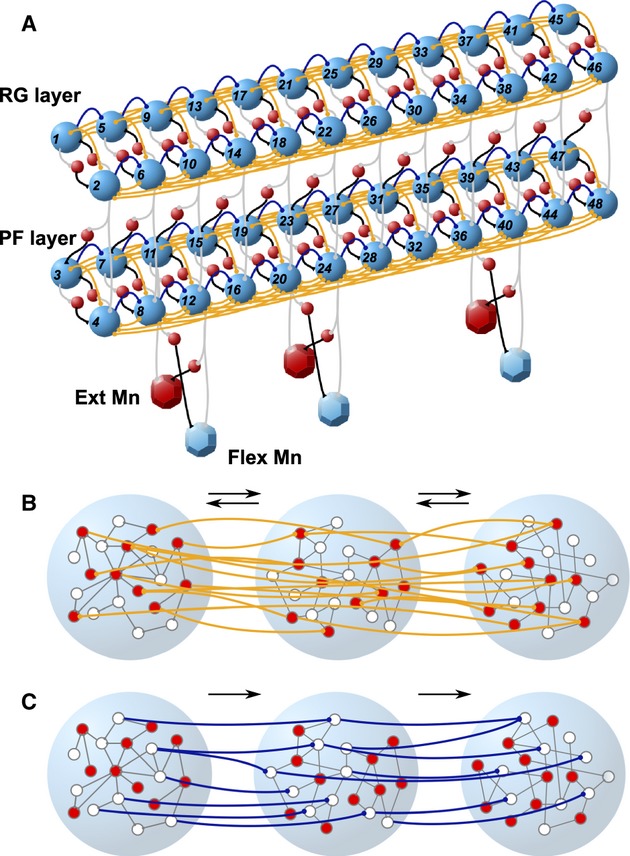
Proposed cat spinal cord network model. (A) Schematic of the proposed intersegmental CPG network. Twelve CPG units were arranged along the cat spinal cord in an asymmetric two-layer organization. Each unit contains excitatory neuronal populations (light blue spheres) and inhibitory interneuronal populations (red spheres) synaptically connected (gray and black lines). Excitatory populations were directly connected by bidirectional connections (orange lines). These CPGs interacted via two types of excitatory connections: bidirectional and feed-forward connections (orange and dark blue lines). (B) Enlargement of three excitatory populations with the bidirectional connectivity in the rostrocaudal direction. In each population, the red and white circles represent spiking and bursting neurons, respectively. These neurons formed a local network with random connectivity (gray lines) with 95% probability. The orange lines represent the bidirectional connections lying only between the spiking neurons. These connections were allowed to connect both rostral and caudal sides until third nearest neighbors in the rostrocaudal direction. In this panel, only bidirectional connections are shown for better visualization, but in our model the feed-forward connections coexisted as shown in (C). (C) The same as (B), but for the feed-forward connections.

To support the hypothesis raised in the experiment (see first paragraph of the previous section and Fig. 1A), we built a spinal cord network model reproducing two experimentally observed phenomena, the sinusoidal electrical wave propagation and the spontaneous zero-lag synchronization embedding two assemblies, as illustrated in Figure 4A. The model consisted of 12 CPGs that covered L4–S1 segments of the spinal cord, following Perez et al. (2009). These CPGs were arranged along the cat spinal cord and interacted via excitatory populations with excitatory synapses. A similar architecture, although based on a single layer, was introduced by Grillner (1981) to account for a rostrocaudal propagation along the spinal cord. This one-layer model, called unit-burst generators, was not sufficient to reproduce some of the observed phenomena during the scratching, such as deletions. The first layer of our model generated the rhythmic movements and controlled their timing, whereas the second layer provided a mechanism to form their patterns (Perret and Cabelguen 1976; Burke et al. 2001; Lafreniere-Roula and McCrea 2005). This two-layer architecture was in agreement with many experimental observations on deletions during the scratching, locomotion, and paw-shake rhythms in cat spinal cords (Perret and Cabelguen 1976, 1980; Duysens 1977; Grillner and Zangger 1979; Duysens and Pearson 1980; Grillner 1981; Perret et al. 1988; Kriellaars et al. 1994; Burke et al. 2001; Kiehn 2006). For these reasons, we adopted the two-layer CPG architecture in our study. The architecture of each CPG formed as follows (See also Fig. 4A): Each CPG unit contained excitatory neurons and inhibitory interneurons in a similar manner as discussed by Brownstone and Wilson (2008). Within each CPG, a half-center rhythm generator (RG) and a pattern formation (PF) network forming a two-layer architecture were considered, as suggested by Rybak et al. (2006). In the RG network two excitatory populations were mutually connected with each other via inhibitory interneuron populations (Fig. 4A, light blue spheres and red small spheres). The excitatory population at the flexor side in the RG directly projected to the excitatory population at the same side in the PF, but to the excitatory population at the extensor side in the PF through an inhibitory interneuron population (the connectivity within a CPG is depicted by gray and black lines in Fig. 4A). The flexor and extensor half-center of the PF network consisted of excitatory populations forming reciprocal connections via inhibitory interneuron populations in the same manner as the RG. The connectivity between the RG and PF was based on the experimental observation that the rhythmic bursting activity of the flexor motoneurons continues during the absence of the extensor bursting activity (Pearson and Iles 1970; Pearson 1972). We assumed, following Brownstone and Wilson (2008), an asymmetric direct excitatory synapse on the flexor PF neuronal population and an interposed inhibitory interneuron population on extensor PF neuronal population. In order to keep the model simple but still realistic, we assumed each population to be composed of 20 neurons. Based on the experimental hypothesis, we embedded two assemblies of neurons into excitatory populations to reproduce the two experimentally observed phenomena, the zero-lag synchronization and the electrical propagation wave. Ermentrout et al. (1998) proposed a similar network model for the Limax olfactory lobe. This model also included both bursting and spiking neurons. The bursting neurons were responsible for the wave generation and the spiking neurons supported the propagation. When removing the connections between bursting and spiking neurons propagation gradient varied, indicating that the spiking neurons played a role of adjusting the wave propagation gradient between the bursting neurons. When the bursting neurons were stimulated, the gradient of the wave propagation collapsed and the model exhibited zero-lag synchronization.

In excitatory populations, we considered 10 neurons of spiking type and the others of bursting type, whereas only bursting type was considered in inhibitory populations. The activity of all neurons in the network was described by the modified Morris–Lecar neuron model (Rinzel and Ermentrout 1998).

To account for the natural heterogeneity in the neurons we randomly distributed the leakage current reversal potential *V*_L_. Within an excitatory population, the neurons were reciprocally connected with 95% random connectivity independent of the neuron type (gray lines in Fig. 4B and C), whereas no connections were assumed in an inhibitory population. Connections between populations within a CPG were set such that each neuron in a population received 90% of randomly chosen neurons from the other population. These apparently large connection strengths were assumed to compensate the small number of neurons in the populations. In addition, we added direct connections to bridge the excitatory populations within the RG and PF (orange lines in Fig. 4A). These connections were assumed only between the spiking neurons in the excitatory populations. The connections between spiking neurons were the same as those of the bidirectional connections in the rostrocaudal direction, which are described below. The CPGs arranged along the cat spinal cord communicated through the excitatory populations with excitatory synapses. There were two types of connectivity between the CPGs: one was the feed-forward connection (Fig. 4A and C, dark blue lines) and the other was the bidirectional connection (Fig. 4A and B, orange lines) that allowed us to embed two assemblies into the network as hypothesized in the experiments described above. These connections linking the GPGs had 85% of the connection probability depending on the neuron types. These connections had axonal conduction delays and their velocity to conduct spike information was taken as 48.6 m/sec (Grottel et al. 1999). To realize the zero-lag synchronization, the mutual connections were restricted to be between the spiking type neurons, and the feed-forward connections were only assumed between the bursting type neurons for the wave propagation. Excitatory synaptic connections from the populations in the PF layer directly excited the motoneurons (see, for instance, connections from the populations 11 and 12 to the extensor and flexor motoneuron populations, respectively, in Fig. 4A). Furthermore, the motoneuron populations were projected from the excitatory populations at the crossing side in the PF layer mediated by inhibitory interneuron populations.

To mimic the neuronal activity of each neuron in the network, we adopted a square-bursting version of the Morris–Lecar model (Rinzel and Ermentrout 1998). The neuron model was given by the following equations:


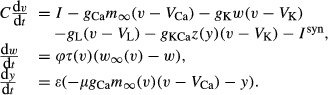


These equations described the evolution in time of the membrane potential, the slow recovery variable, and the calcium concentration. The variable *C* represented the membrane capacitance per unit of area. This conductance-based model described the dynamics of the membrane potential, assuming four ionic currents: the calcium, potassium, leakage, and calcium-dependent potassium channels with conductivities given by *g*_Ca_, *g*_K_, *g*_L_, and *g*_KCa_, respectively. The incorporation of the calcium-dependent potassium channel allowed the model to qualitatively reproduce the bursting behavior as observed in the experiments. The parameter *φ* represented the difference in timescales between the membrane potential and the recovery variable. The parameters *μ* and *ε* governed and controlled the ionic channel dynamics. The parameter *μ* determined the ratio of the calcium volume to the surface area of the cell. The parameter *ε* was the product of the calcium degradation rate and the ratio of free to total calcium. This parameter is typically set to a small value because calcium is usually neutralized, so the variable *y* has slow dynamics relative to the variable *v*. By adjusting these two parameters, we could control the duration and period of the bursts. The model did not take into account the sodium channels, resulting in a reasonable agreement with experimental observations. The nonlinear functions controlling the dynamics of ionic currents were given by


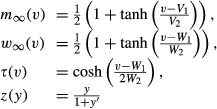


where *V*_1_, *V*_2_, *W*_1_, and *W*_2_ were constants.

The synaptic current of each neuron was described by the following equation:


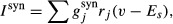


where 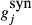 represented the maximum conductance of the *j*th presynaptic channel, *E*_s_ the reversal potential, and the kinetics *r*_*j*_ of bound receptors was described as:


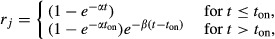


where *α* and *β* were the rise and decay rate constants for transmitter binding, and *t*_on_ was a time constant determining the shifting point from the rise to the delay of the synaptic kinetics (Destgexhe et al. 1994).

All the parameters for the neuron model and the synaptic connectivity used in the simulations are listed in Tables 1, 2, respectively. All neurons in the network were stimulated by a weak noisy external input following a Poisson distribution as the stimulation of low-threshold cutaneous and Ib muscle afferents. During the wave propagation phase, we increased the applied current for the bursting neurons in the populations 9, 10, 11, and 12 in both RG and PF to *I* = 43.8 *μ*A/cm^2^ in order to evoke the incoming stimulations into the network, whereas the applied current to the remaining bursting neurons in the populations were kept being the constant value just below the spiking threshold.

**Table 1:** Parameters for neurons

Parameter	Value for bursting neurons	Value for spiking neurons
*C*	5 μF/cm^2^	20 μF/cm^2^
*g*_Ca_	4 mS/cm^2^	4 mS/cm^2^
*g*_K_	8 mS/cm^2^	8 mS/cm^2^
*g*_L_	2 mS/cm^2^	2 mS/cm^2^
*g*_KCa_	0.25 mS/cm^2^	0 mS/cm^2^
*V*_Ca_	120 mV	120 mV
*V*_K_	−84 mV	−84 mV
*V*_L_	−60 **±** 0.01 mV	−60 **±** 0.01 mV
*V*_1_	1.2 mV	1.2 mV
*V*_2_	18 mV	18 mV
*W*_1_	12 mV	12 mV
*W*_2_	17.4 mV	17.4 mV
*ϕ*	0.92 sec^**−**1^	1/15 sec^−1^
*ε*	0.0175 sec^**−**1^	0 sec^−1^
*μ*	0.0149–0.015 sec^−1^	0 sec^−1^
*I*	43 *μ*A/cm^2^	39.7**–**39.9 *μ*A/cm^2^

**Table 2:** Parameters for synapses

Parameter	Value
*α*	0.33 msec^**−**1^
*β*	0.1 msec^−1^
*t*_on_	1 msec
*E*_s_	0 (excitatory), −80 (inhibitory) mV

To account for the experimental observations (Manjarrez et al. 2003), we allowed the spiking neurons in the network to have different firing rates by varying the applied current value *I* in the equation for *v*. The variable *I* was assumed to be uniformly distributed in the range between 39.7 and 39.9 *μ*A/cm^2^. Similarly, we varied the parameters *μ* of the bursting neurons in the equation for *y* to change the bursting duration as observed in experiments. For neurons belonging to populations at the extensor side in Figure 4A, for example, populations 9 and 11, the extensor motoneuron population, we set *μ* to 0.015 that led their bursting duration to ∼140 msec, whereas neurons at the flexor side, for example, the populations 10 and 12, the flexor motoneuron population, had *μ* = 0.0149 that made their bursting duration ∼70 msec as observed experimentally (Cuellar et al. 2009; Perez et al. 2009; see also Bonnot et al. 2002; Yakovenko et al. 2002; Kaske et al. 2003; Ivanenko et al. 2006; Falgairolle and Cazalets 2007).

All the synaptic strengths were characterized by the maximum conductances. As there were two types of neurons within an excitatory population, there were four types of connections (Fig. 4B and C, gray lines). We described *g*_bur→bur_, *g*_bur→spk_, *g*_spk→bur_, and *g*_spk→spk_ as the maximum conductances of these connections (Table 3). The subscripts “spk” and “bur” stood for the excitatory spiking and bursting neurons, respectively. In a CPG, five types of connections enabled the neurons between pairs of populations to interact with each other (Fig. 4A, gray and black lines). The synaptic connections among these neurons had the strengths of *g*_bur→ex_, *g*_spk→ex_, *g*_bur→inh_, *g*_spk→inh_, and *g*_inh→ex_ (Table 4). The subscripts “ex” and “inh” indicated excitatory and inhibitory neurons. Here, “ex” represented the fact that any types of neurons, bursting or spiking neuron, were possible. For instance, *g*_bur→ex_ indicates that there were both *g*_bur→bur_ and *g*_bur→spk_, but *g*_bur→bur_ = *g*_bur→spk_. The arranged CPGs interacted through the excitatory populations with the two types of connections: feed-forward and bidirectional connections (Fig. 4, dark blue and orange lines). The strengths of these connections were indicated as *g*_ff_ and *g*_bb_ (Table 5).

**Table 3:** Coupling strengths within an excitatory population

Parameter	Value
*g*_bur→bur_	0.1 mS/cm^2^
*g*_bur→spk_	0.1 mS/cm^2^
*g*_spk→bur_	0.01 mS/cm^2^
*g*_spk→spk_	0.1 mS/cm^2^

**Table 4:** Coupli strengths between two populations within a CPG

Parameter	Value
*g*_bur→ex_	0.1 mS/cm^2^
*g*_spk→ex_	0.01 mS/cm^2^
*g*_bur→inh_	0.12 mS/cm^2^
*g*_spk→inh_	0.01 mS/cm^2^
*g*_inh→ex_	0.025–0.05 mS/cm^2^

**Table 5:** Other coupling strengths

Parameter	Value
*g*_bid_	0.12 mS/cm^2^
*g*_ff_	0.1 mS/cm^2^
*g*_ext_	0.1 mS/cm^2^

**Figure 5:**
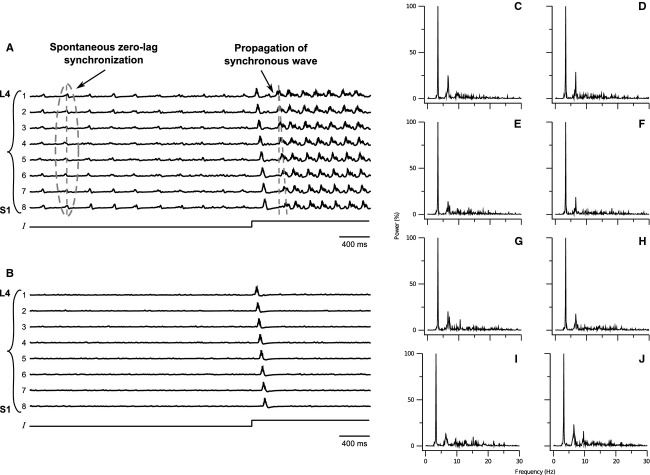
Spontaneous zero-lag synchronization between intersegments and the propagation of the electrical synchronous wave along the cat spinal cord. (A) Time traces of the mean potentials of PF layers in CPGs. The number from one to eight corresponds to the CPG unit from third to tenth. (B) Same as A, but with parameters *g*_bid_ setting to zero. (C–J) Power spectra during zero-lag synchronization phase. The power in the spectra is normalized by the maximum value. (C–J) were, respectively, obtained from the time traces before the stimulus to the bursting neurons were increased.

**Figure 6:**
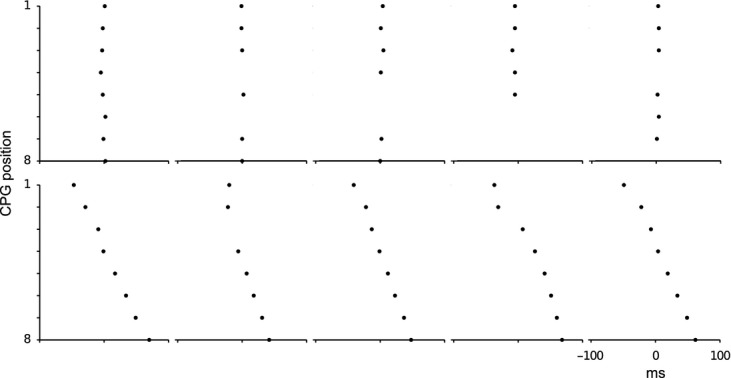
Raster display of spontaneous zero-lag synchronization and electrical wave propagation. The upper five raster plots are shown in two alternative phenomena. The points correspond to peaks of the mean potentials in the absence of the incoming stimulations in Figure 5A. The lower five raster plots are for the traveling wave propagation after the increase in the biased current in Figure 5A. For the detection of the peaks, we applied low-pass filter to the data with a cut-off frequency of 4.3 Hz. In the zero-lag synchronization raster displays, the synchronous activities among CPGs are plotted in which the temporal resolutions are higher than 10 msec.

We computed the mean potential of excitatory populations in the RG layer in the set of third (more rostral) to tenth (more caudal) CPG units that corresponded to the electrodes 17–24 or 9–16 in the experiments, in the absence of the incoming stimulation (Fig. 5A). In Figure 5A, these CPGs were indexed from 1 to 8 to compare with the experimental data shown in Figures 2, 3. Before the step current was applied to the excitatory bursting neurons in the third CPG, the mean potentials of all the observed units fluctuated due to the weak noisy input. Although the mean potentials exhibited a noisy behavior, some sharp peaks were observed in the trajectory of the mean potential, indicating that neurons in a CPG unit synchronously fired. In addition, such synchronous activities sometimes occurred at the same time among intersegments (Fig. 5A, gray dashed line circle). The synchronization among intersegments composed of only spiking neurons was derived only from the connectivity between spiking neurons. Before increasing the biased current, all neurons in the network were stimulated by the weak external noisy input (Table 5, *g*_ext_). However, most of the bursting neurons remained producing subthreshold activity because their bias current was set well below their firing threshold as compared to the spiking neurons. Consequently, only spiking neurons exhibited firings under such current. Because these spiking neurons were stimulated by the weak noisy input, they fired sparsely and randomly if there were no bidirectional connections (Fig. 5A and B, orange lines) among them. Indeed if the bidirectional connections (Fig. 5A and B, orange lines) were removed, namely, if *g*_bid_ = 0, the synchronous firings of the neuronal assemblies were absent as seen in Figure 5B, indicating that the spiking neurons fired asynchronously. From these results, although there were other possibilities, our assumption of the bidirectional connections between the spiking neurons was one of the possible mechanisms to realize the zero-lag synchronization in the cat spinal cord, as was suggested in thalamocortical circuits as well (Gollo et al. 2010). For easy comparison, we also plotted peaks of the mean potential in the zero-lag synchronization (Fig. 6 top panels). Analogous to the experimental results (Fig. 2), highly precise synchronization among intersegments was observed.

For further analysis, we also carried out the spectrum analysis during the zero-lag synchronization phase and the power spectra of individual segments, which are shown in Figure 5C–J. All the spectra had characteristic frequency components until 20 Hz as observed in the experiments conducted by Manjarrez et al. (2002). In their analysis, the largest peak was basically located below 5 Hz, although some peaks were exhibited until 20 Hz. The model qualitatively captured the characteristic frequency components observed in the experiments.

The mean potentials of spinal segments, after increasing the biased current of the bursting neurons, oscillated as shown in Figure 5A. In this phase, although neurons within each segment exhibited synchronous firings, the synchronization was rhythmical. Additionally, these synchronizations did not happen among intersegments simultaneously. Instead of the zero-lag synchronization, the synchronization within each segment consecutively appeared in the rostrocaudal direction, indicating that the synchronous activity in each segment propagated along the cat spinal cord. The raster plot of the peak of the mean membrane potential clearly showed this propagation (Fig. 6 bottom panels). The mechanism to generate the sinusoidal electrical wave propagation was same as the one used in the model proposed by Perez et al. (2009) as our model inherited the network architecture from the previous model to induce the wave propagation. In brief, the bursting neurons belonging to the third segment were activated by the increase in their biased currents. These bursting neurons began to fire simultaneously because the biased current increased at the same time, but after some transient the bursting neurons at the extensor side fired in antiphase with those at the flexor side because of the half-center RG or PF. These firings were conducted through the feed-forward connections (Fig. 4A and C, dark blue lines) and strongly stimulated the bursting neurons in the next segment. Because the biased current was set just below their firing thresholds, the bursting neurons in the next segment fired. Their firings therefore delayed from the firings in the previous segment. By repeating this procedure, the sinusoidal electrical wave was propagated along the cat spinal cord. We also performed numerical simulations of the network without the bidirectional connections (Fig. 4A and B) lying among the spiking neurons (Fig. 5B). Interestingly, we observed that not only the zero-lag synchronization among intersegments but also the electrical wave propagation disappeared by pruning of the bidirectional connections. The pruning of these connections, that reduces the synchronous activity of the spiking neurons, resulted in decreasing the baseline of the membrane potentials of the bursting neurons as well because the bursting neurons locally interacted with the spiking neurons in individual excitatory populations. For this reason, the stable propagation of the synchronization disappeared, suggesting that the zero-lag synchronization among intersegments played an important role to support or regulate the stable electrical wave propagation during the fictive scratching or the locomotion in the cat spinal cord.

## Discussion

In previous studies, some characteristics of both the spontaneous nCDPs (Manjarrez et al. 2000; Manjarrez et al. 2002) and the sinusoidal CDPs (Cuellar et al. 2009; Perez et al. 2009) were separately analyzed. Based on those results, the purpose of this study was to compare the simultaneous occurrence of these electrical field potentials. Our study suggests that the spontaneous nCDPs and the sinusoidal CDPs could be generated by two different neuronal groups; although, we do not exclude other neuronal configurations. On the one hand, the spontaneous activity was produced by dorsal horn neurons that responded monosynaptically to stimulation of low-threshold cutaneous afferents. These neurons are involved in mechanisms mediating the primary afferent depolarization (PAD) of muscle and cutaneous afferents and appear to be a source of variability of Ia monosynaptic reflexes. The full understanding of the physiological role of the spontaneous activity is not clear yet, but these dorsal horn neurons could play a role in the regulation of the transmission of sensory and motor pathways. An interesting observation derived from Cuellar et al. (2009) is that the spontaneous nCDPs were not in phase or rhythmically produced during the fictive scratching. The latter suggests that in the lumbar spinal cord the neurons belonging to the CPG and the neurons producing the spontaneous nCDPs are of different populations. Further details about a possible modulation between the neuronal networks are still lacking. In this sense, studies on the firing profile of spinal neurons related to the spontaneous nCDPs active during a fictive motor task are suitable to promote and inspire new models. It would be also interesting to study the firing profile of the neurons that are rhythmically active during the cycles of a fictive motor task (e.g., scratching or locomotion) and to analyze if this firing is related with the spontaneous activity. In this context, the presented results suggest two different neuronal populations; that is, the circuitry of these neuronal networks stands for different architectural elements. Otherwise, despite the latest efforts trying to elucidate the motor circuits, there is no agreement about the architecture of the CPGs, but the theoretical models supported by the experimental data offer the advantage of reproducing and establishing some proposals and hypothesis (see for example McCrea and Rybak 2008; and Perez et al. 2009). A two-layer CPG architecture has been proposed because this organization is consistent with many experimental observations on deletions during scratching and locomotion in the cat (Lafreniere-Roula and McCrea 2005; Perez et al. 2009). The first layer represents the rhythm generation and controls the timing, whereas the second layer provides a mechanism for the PF.

### Experimental validation for the zero-lag synchronization

This study is also important because it strongly suggests that the zero-lag synchronization is not an epiphenomenon due to the volume conduction. This suggestion is based on the experimental and theoretical observation that the same longitudinally distributed electrodes can simultaneously detect synchronization and propagation of electrical potentials of similar amplitude. Moreover, if the synchronization of the electrical nCDPs was produced by the spontaneous activation of a single neuronal group located in one restricted region of the spinal cord with electrical propagation throughout the volume conduction, then it would be not possible to observe desynchronized nCDPs in in-situ “isolated” dorsal horn spinal segments after the spinal transversal section of the dorsal horn and the dorsolateral funiculus (Manjarrez et al. 2003). It was observed that after the dorsal horn sectioning, the nCDPs of similar amplitude were still recorded but they were desynchronized. This suggests that the synchronization of the longitudinally distributed nCDPs is not due to volume conduction properties and it is generated by the synaptic interconnections between neuronal ensembles in the spinal cord. In this study we suggest a plausible explanation of these observations: we propose that a zero-lag synchronization mechanism is involved, as shown by our modeling results.

These observations in the cat spinal cord provide another example of zero-lag synchronization among distant areas. In the early 1990s experimental studies also reported zero-lag synchronization between different cortical areas of the brain (Fries et al. 1997; Roelfsema et al. 1997). To explain this zero-lag synchronization, Ermentrout and Kopell (1998) developed a model in terms of a canonical circuit of excitatory and inhibitory neurons. More recently, a model of two neuronal populations coupled through a relay element was also proposed. It was found that, even in the presence of large axonal conduction delays, distant neuronal populations self-organize into lag-free oscillations (Vicente et al. 2008). It is quite probable that different mechanisms are responsible for bringing synchrony at different levels. Network structure and the participation of different cerebral structures must influence in the synchronization. For instance, R. Llinas and other authors have suggested that the reciprocal coupling of cortical areas with the thalamus as a mechanism to support distributed cortical processing and the emergence of consciousness (Llinas and Pare 1997; Llinas et al. 1998; Sherman and Guillery 2002). Also an experimental work by Contrera and coworkers supported the participation of the thalamus as a deep coronal cut, through the suprasylvian gyrus, was observed not to disturb the synchrony of spindle oscillations across regions of the cortex located at both sides of the lesion (Contreras et al. 1996). Based on these observations, it was studied, and indeed suggested numerically that the thalamus could play the role of relay element mediating in the synchronized dynamics between two cortical areas (Gollo et al. 2010). Similar behaviors were observed, both experimentally and in a model, when the hippocampus mediates between two cortical areas (Gollo et al. 2011).
